# CD71^+^ Erythroid Cell Expansion in Adult Sepsis: Potential Causes and Role in Prognosis and Nosocomial Infection Prediction

**DOI:** 10.3389/fimmu.2022.830025

**Published:** 2022-02-18

**Authors:** Guang-ju Zhao, Dan-wei Jiang, Wen-chao Cai, Xiao-Yan Chen, Wei Dong, Long-wang Chen, Guang-liang Hong, Bin Wu, Yong-ming Yao, Zhong-qiu Lu

**Affiliations:** ^1^ Emergency Intensive Care Unit, Emergency Department, The First Affiliated Hospital of Wenzhou Medical University, Wenzhou, China; ^2^ Trauma Research Center, Fourth Medical of the Chinese People’s Liberation Army (PLA) General Hospital, Beijing, China

**Keywords:** sepsis, nosocomial infections, CD71^+^ erythroid cells, erythroid progenitors, IL-6

## Abstract

**Background:**

Immune suppression contributes to nosocomial infections (NIs) and poor prognosis in sepsis. Recent studies revealed that CD71^+^ erythroid cells had unappreciated immunosuppressive functions. This study aimed to investigate the values of CD71^+^ erythroid cells (CECs) in predicting NIs and prognosis among adult septic patients. The potential factors associated with the expansion of CECs were also explored.

**Methods:**

In total, 112 septic patients and 32 critically ill controls were enrolled. The frequencies of CD71^+^ cells, CD71^+^CD235a^+^ cells, and CD45^+^ CECs were measured by flow cytometry. The associations between CECs and NIs and 30-day mortality were assessed by ROC curve analysis and Cox and competing-risk regression models. Factors associated with the frequency of CECs were identified by linear regression analysis.

**Results:**

The percentage of CD71^+^ cells, CECs, and CD45^+^ CECs were higher in septic patients than critically ill controls. In septic patients, the percentages of CD71^+^ cells, CECs, and CD45^+^ CECs were associated with NI development, while CD71^+^ cells and CECs were independently associated with 30-day mortality. Linear regression analysis showed that the levels of interleukin (IL)-6 and interferon (IFN)-γ were positively associated with the frequencies of CD71^+^ cells, CECs, and CD45^+^ CECs, while IL-10 was negatively associated with them. Additionally, the levels of red blood cells (RBCs) were negatively associated with the percentage of CD45^+^ CECs.

**Conclusions:**

CECs were expanded in sepsis and can serve as independent predictors of the development of NI and 30-day mortality. Low levels of RBCs and high levels of IL-6 and IFN-γ may contribute to the expansion of CECs in sepsis.

**Trial Registration:**

ChiCTR, ChiCTR1900024887. Registered 2 August 2019, http://www.chictr.org.cn/showproj.aspx?proj=38645

## Introduction

Sepsis and septic shock are major healthcare problems worldwide, and mortality and morbidity are still high (1). Sepsis is often accompanied by immune suppression, which is associated with increased occurrence of nosocomial infection (NI) and adverse outcomes (2, 3). The immunosuppression of sepsis is characterized by the insufficient response of immune cells, decreased number of immune cells, increased level of anti-inflammatory cytokines, and the expansion of immunosuppressive cells (4, 5). It is generally acknowledged that immunosuppression mainly occurs in the late stage of sepsis, but recent evidence shows that this phenomenon also exists in the early stage of the disease and is closely related to the poor prognosis of patients (5–7).

Erythropoiesis is a highly regulated, multistep process that generates mature red blood cells (RBCs) from hematopoietic stem cells (HSCs) in bone marrow. During anemia, pregnancy, or infections, extramedullary erythropoiesis is induced in the spleen and a large number of immature erythrocytes with immunosuppressive function were produced (8, 9). Although immunophenotype patterns for these erythroid cells are not totally identified, transferrin receptor I (CD71) combined with glycophorin A (CD235a) is widely used to characterize them in humans (8–10). Recently, two subtypes of CD71^+^ erythroid cells (CECs) with different immunosuppressive abilities were identified on the basis of differential expression of CD45. Compared to CD45^+^ CECs, some studies showed that CD45^-^ CECs have relatively poor immunosuppressive properties. However, in animal models, transfer of CD45^-^ CECs also promotes tumor growth, indicating they may also have immunomodulatory activities (10–12).

In neonates, the relationship between the expansion of CECs and numerous pathogen infections has been well described. CECs from newborns suppressed cytokine production in myeloid cells and T cells (13). Additionally, CEC-deficient neonatal mice are more resistant to pathogens including *Listeria monocytogenes*, *Escherichia coli*, and *Bordetella pertussis* (14, 15). However, the frequency of CECs in adult critical illness patients with sepsis and its clinical significance remains unclear. In this study, we evaluated whether CECs can be used to evaluate the risk of nosocomial infection and the prognosis of adult septic patients. Furthermore, the potential factors associated with the expansion of CECs in sepsis were also explored.

## Methods

### Patients

The study was conducted at an 18-bed emergency intensive care unit (ICU) of the First Affiliated Hospital of Wenzhou Medical University, which is a tertiary hospital of City University, with about 300,000 emergency admissions every year. The study was reviewed and approved by the Institutional Review Board of the First Affiliated Hospital of Wenzhou Medical University, Wenzhou, China (2019040), and was registered on the website of China Registered Clinical Trial Registration Center with No. ChiCTR1900024887. Informed consent was obtained from all participants prior to enrollment.

Between July 15, 2019 and August 15, 2020, patients with sepsis and septic shock were prospectively enrolled in our study. Sepsis was defined as a documented infection and an acute increase of 2 SOFA points according to the diagnostic criteria of Sepsis-3 (16). Patients aged <18 years or with acute bleeding were excluded. Patients were excluded from the study if they received erythropoietin or stored RBCs during hospitalization or within 3 months before admission.

### Data Collection and Definitions

Baseline data collection included demographics (age and gender), height, weight, comorbidities, source of sepsis, and nosocomial infections. Septic patients were screened daily for nosocomial infection which was defined as a new infection acquired more than 48 h after admission. The diagnosis of nosocomial infection in the present study was made based on the criteria of Centers for Disease Control and Prevention (CDC, 2008), as we previously described (3, 17, 18). Clinical scores, including Acute Physiology and Chronic Health Evaluation (APACHE) II and Sepsis Related Organ Failure Assessment (SOFA) score, were also recorded in the first 24 h after admission. Length of stay in hospital and ICU and the outcome after 30 days (death or survival) were collected.

### Laboratory Examinations

The Sysmex XE-2100 automatic blood cell analyzer was used to detect the levels of white blood cells (WBCs), red blood cells (RBCs), hemoglobin (Hb), and lymphocytes. The levels of serum creatinine (Scr) were tested using the Vitros 5.1 FS Chemistry System (Ortho Clinical Diagnostics, Johnson & Johnson, New Brunswick, NJ, USA). Cytokines including interleukin (IL)-2, IL-4, IL-6, IL-10, tumor necrosis factor (TNF)-α, and interferon (IFN)-γ in serum were detected by a microsphere-based immunofluorescence assaying kit (Saiji Biotechnology, Jiangxi, China).

### Flow Cytometry

Samples of peripheral blood were collected from patients within 2 days after admission and transported to the laboratory at 4°C immediately. Peripheral blood mononuclear cells (PBMCs) were isolated from whole blood through Ficoll centrifugation. Specifically, the following antibodies were used: FITC-labeled anti-CD71 and its isotype control antibody (Ab) (IgG2a, κ), APC-labeled anti-CD235a and its isotype control Ab (IgG2b, κ), and PE-labeled anti-CD45 and its isotype control Ab (IgG1, κ). All antibodies were purchased from BD Biosciences Company (San Jose, CA, USA). The frequency of cells was analyzed by flow cytometry on a BD FACSCanto II flow cytometer (BD Biosciences). Fluorescence minus one (FMO) isotype control was used to confirm the specificity of staining.

### Data Analysis and Statistics

Continuous variables in the present study were described as a median with interquartile range or mean ± SD. Categorical variables were reported as frequencies. Univariate analyses were conducted using the Mann–Whitney U test or Kruskal–Wallis H test for continuous variables, chi-square test for categorical date. The Cox regression model was used for estimating the relationships between CECs and outcomes (NI and mortality) adjusting for confounding variables selected based on the results of univariate analysis. To determine the discriminative power of the variables for NI and mortality, we constructed ROC curves and calculated areas under the curve (AUC) with 95% CI. The best predictive cutoff values maximizing the sum of sensitivity and specificity were defined. Further, cumulative incidence curves were used to compare the incidence of NI and 30-day mortality among septic patients according to these cutoff values. Competing risk regression models were used to assess the risk of NI and 30-day mortality. Multiple linear regression was performed to detect independent variables associated with the frequency of CD71^+^ cells and CECs. In all tests, two-tailed p < 0.05 was considered significant. The calculations were performed with IBM SPSS Statistics version 22.0 (IBM, Armonk, NY, USA), GraphPad Prism (GraphPad Software) and R 3.6.2 software for Windows.

## Results

### Characteristics of Study Subjects

**Figure 1:**
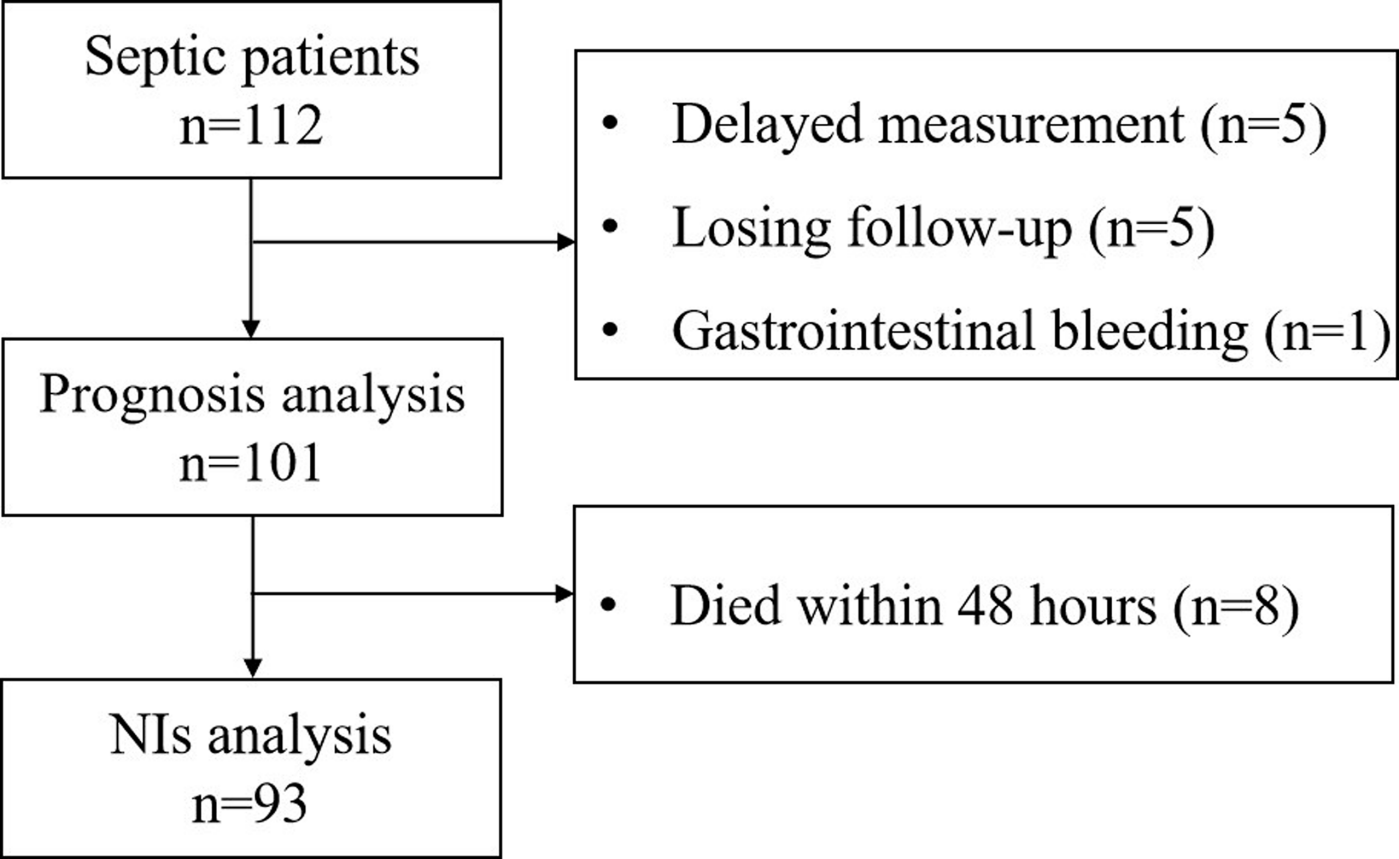
Flowchart of the included patients and reasons for patient exclusion.

Initially, 112 septic patients were admitted to the ICU of the emergency department. However, five septic patients had a delayed measurement. Five patients were discharged automatically and lost to follow-up. One patient with gastrointestinal bleeding was excluded. Thus, 101 septic patients were included in the survival analysis. A total of 32 critically ill patients without sepsis were enrolled as control. The flow diagram of selecting study subjects is shown in **Figure 1**. The demographic and clinical characteristics of patients are shown in **Table S1**. There were no differences in baseline characteristics between the septic patients and critically ill patients without sepsis on admission (**Table S1**).

### Nosocomial Infection Characteristics

For NI analysis, 8 septic patients who died within 48 h after admission were excluded, and 93 patients were enrolled finally (**Figure 1**). Twenty-nine percent (27/93) of patients developed NIs. Of these, 23 patients had an NI at one site, and 5 patients had NIs at two sites. The median time of the first diagnosis of NIs was 8 days [interquartile ranges (IQR): 4–15]. Among 33 NIs, pulmonary infection (PI) was the most frequent NI (60.6%), followed by urinary tract infection (UTI) (21.2%), bloodstream infection (BSI) (9.1%), and catheter-related infections (CRI) (9.1%). Of all NIs, 39 microorganisms were isolated. PIs were mostly caused by *Acinetobacter baumannii* and *Stenotrophomonas maltophilia*, followed by *Pseudomonas aeruginosa*, *Burkholderia cepacia*, and *Corynebacterium*. Candida was the most common pathogen responsible for UTIs and CRIs. Staphylococcus accounted for 66.7% of all isolates from BSIs. Details regarding the frequency of isolated microorganisms are given in **Table S2**.

The baseline characteristics of patients with and without NI are shown in **Table 1**. There were no significant differences in age, gender, and comorbidities between patients that will develop NI or not. Septic patients who developed an NI were more severely ill on admission than those who did not develop an NI, as indicated by higher APACHE II score and SOFA score. Moreover, these patients were characterized by greater exposure to intubation and central venous catheterization, and longer duration of mechanical ventilation, and urinary tract and central venous catheterization. The 30-day mortality was significantly higher in patients with NI (p = 0.034) (**Table 1**).

**Table 1:** Baseline characteristics of septic patients with and without NIs.

Variables	Septic Patients			p value
	Total (n = 93)	Without NI (n = 66)	With NI (n = 27)	
Age (year)	63.8 ± 1.2	63.6 ± 1.6	64.2 ± 2.0	0.744
Gender (male), n(%)	50(53.8%)	34(51.5%)	16(59.3%)	0.327
BMI, kg/m^2^	22.9 ± 0.4	23.2 ± 0.5	21.9 ± 0.8	0.125
Percentage of CD71^+^ cells	2.9 (1.2, 6.4)	2.5 (1.1, 5.7)	4.0 (1.4, 12.6)	0.113
Percentage of CD71^+^CD235a^+^ cells	0.9 (0.2, 1.9)	0.7 (0.2, 1.3)	1.2 (0.5, 4.1)	0.021
Percentage of CD45^+^CD71^+^CD235a^+^ cells	0.10 (0.04,0.19)	0.08 (0.04,0.17)	0.15 (0.07,0.36)	0.016
Circulating cytokines (pg/ml)				
IL-2	0.7 (0.5, 1.2)	0.7 (0.5, 1.2)	0.6 (0.5, 1.4)	0.879
IL-4	0.4 (0.1, 0.6)	0.4 (0.1, 0.5)	0.3 (0.2, 0.7)	0.549
IL-6	267.1 (72.1, 2971.3)	181.1 (66.4, 2971.3)	922.9 (105.3, 3000.1)	0.139
IL-10	44.1 (12.2, 398.1)	43.2 (13.2, 407.7)	50.7 (7.6, 313.2)	0.997
TNF-α	1.8 (0.7, 5.6)	1.8 (0.7, 5.6)	1.5 (0.5,5.6)	0.836
IFN-γ	1.6 (0.5, 3.2)	2 (0.6, 3.2)	1 (0.1, 4.8)	0.381
Co-morbidities, n (%)				
Diabetes	20 (21.5%)	13 (19.7%)	7 (25.9%)	0.343
Chronic heart disease	9 (9.7%)	5 (7.6%)	4 (14.8%)	0.240
Hypertension	50 (53.8%)	37 (56.1%)	13 (48.1%)	0.320
Chronic liver disease	8 (8.6%)	4 (6.1%)	4 (14.8%)	0.167
Chronic kidney disease	12 (12.9%)	9 (13.6%)	3 (11.1%)	0.519
Cancer	17 (18.3%)	11 (16.7%)	6 (22.2%)	0.361
Infection sites, n (%)				0.262
Digestive tract	30 (32.3%)	19 (28.8%)	11 (40.7%)	
Urinary tract	25 (26.9%)	21 (31.8%)	4 (14.8%)	
Skin and soft tissue	15 (16.1%)	9 (13.6%)	6 (22.2%)	
Respiratory tract	7 (7.5%)	4 (6.1%)	3 (11.1%)	
Others	16 (17.2%)	13 (19.7%)	3 (11.1%)	
Pathogen isolates, n (%)				0.036
Gram-negative bacteria	19 (20.4%)	15 (22.7%)	4 (14.8%)	
Gram-positive bacteria	9 (9.7%)	5 (7.6%)	4 (14.8%)	
Fungus	2 (2.2%)	1 (1.5%)	1 (3.7%)	
Virus	1 (1.1%)	0 (0%)	1 (3.7%)	
^a^Other pathogens	3 (3.2%)	3 (4.5%)	0 (0%)	
Mixed	3 (3.2%)	0 (0%)	3 (11.1%)	
No	56 (60.2%)	42 (63.6%)	14 (51.9%)	
^b^SOFA score, median (IQR)	7 (4, 9)	6 (4, 9)	9 (7, 12)	0.002
^b^APACHE II score				
Overall, median (IQR)	13 (9, 17)	12 (8, 15)	17 (13, 20)	0.001
<8	21 (22.6%)	17 (25.8%)	4 (14.8%)	0.005
9–13	26 (28%)	23 (34.8%)	3 (11.1%)	
14–17	25 (26.9%)	17 (25.8%)	8 (29.6%)	
>17	21 (22.6%)	9 (13.6%)	12 (44.4%)	
Hemoglobin (g/L)	103.0 (86.5, 116.0)	105.0 (90.8, 118.0)	97.0 (71.0, 112.0)	0.069
Red blood cells (×10^12/L)	3.5 (2.9, 3.9)	3.5 (3, 3.9)	3.5 (2.6, 3.8)	0.345
White blood cells (×10^9/L)	13.9 (8.2, 20.3)	14.5 (8.3, 19.9)	12.4 (7.6, 21.2)	0.651
Lymphocytes (×10^9/L)	0.7 (0.5, 1.0)	0.7 (0.5, 1)	0.7 (0.5, 1)	0.343
Serum creatinine (μmol/L)	136.0 (83.5, 266.5)	111.5 (76.8, 219.0)	214.0 (102.0, 296.0)	0.048
Interventions, n (%)				
Intubation	22 (23%)	6 (9.1%)	16 (59.3%)	<0.001
Duration of mechanical ventilation	0 (0, 0)	0 (0, 0)	3 (0, 11)	<0.001
Central venous catheterization	65 (69.9%)	43 (65.2%)	22 (81.5%)	0.093
Duration of central venous catheterization	4.0 (0, 8.0)	3 (0, 6)	9 (3, 15)	<0.001
Urinary tract catheterization	87 (93.5%)	61 (92.4%)	26 (96.3%)	0.436
Duration of urinary tract catheterization	6.0 (4.0, 11.5)	5 (3, 7)	12 (7, 26)	<0.001
30-day mortality, n (%)	13 (14.0%)	6 (9.1%)	7 (25.9%)	0.034

NI, nosocomial infection; IQR, interquartile ranges; APACHE, Acute Physiology and Chronic Health Evaluation; SOFA, Sequential Organ Failure Assessment.

a Other pathogens are rickettsia, leptospira, and plasmodium falciparum.

b Scores were calculated within the first 24 h after ICU admission using the value associated with the greatest severity of illness.

### CD71^+^ Cells and CECs in Different Groups

**Figure 2:**
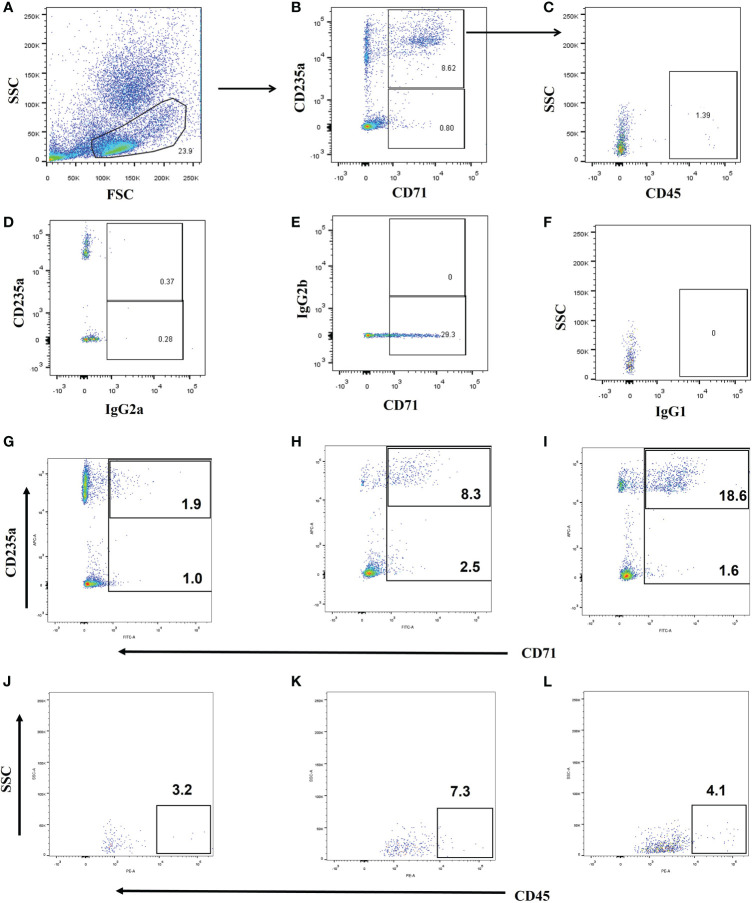
Gating strategy and representative flow cytometry plots. (1) Gating strategy for CD71^+^ cells, CD71^+^CD235a^+^ cells, and CD71^+^CD235a^+^CD45^+^ cells **(A–C)**. CD71^+^CD235a^-^ cells, and CD71^+^CD235a^+^ cells were gated from peripheral blood mononuclear cells (PBMCs), and then CD71^+^CD235a^+^CD45^+^ cells were gated from CD71^+^CD235a^+^ cells. The total percentages of CD71^+^ cells, CD71^+^CD235a^+^ cells, and CD71^+^CD235a^+^CD45^+^ cells were calculated and recorded, respectively. (2) Fluorescence minus one (FMO) isotype control for proper identification of CD71^+^ **(D)**, CD235a^+^ **(E)** and CD45^+^ cells **(F)**. (3) Representative flow cytometry plots for the assessment of CD71^+^ cells, CD71^+^CD235a^+^ cells, and CD71^+^CD235a^+^CD45^+^ cells in ICU control **(G, H)**, septic patients **(I, J)**, and septic patients who had nosocomial infection and died **(K, L)**.

**Figure 3:**
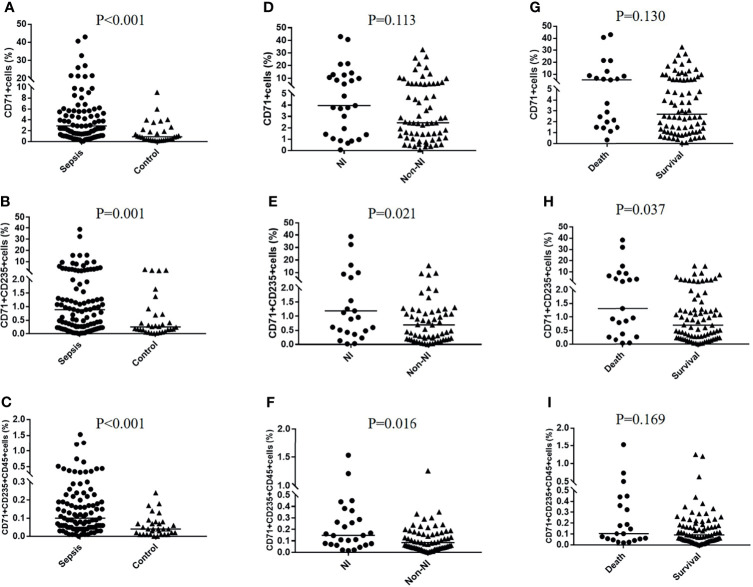
CD71^+^ cells, CECs, and CD45^+^CECs in different groups. The percentages of CD71^+^ cells, CD71^+^CD235a^+^ cells, and CD71^+^CD235a^+^CD45^+^ cells were compared between the sepsis group and control group **(A–C)**, nosocomial infection (NI) group and non-NI group **(D–F)**, and death group and survival group **(G–I)**, respectively.

Representative flow dot plots of gating strategies of CD71^+^ cells, CD71^+^CD235a^+^ cells, and a CD45^+^ subset of CD71^+^CD235a^+^ cells are shown in **Figure 2**. Their frequency varies greatly among adult critically ill patients; the proportion of CD71^+^CD235^+^ cells in PBMC ranged from below a detectable level to 38.80% (**Figure S1**). Compared with critically ill controls, the percentages of CD71^+^ cells, CD71^+^CD235a^+^ cells, and CD71^+^CD235a^+^CD45^+^ cells in PMBCs were significantly higher in septic patients (all p < 0.01) (**Figures 3A–C**). Septic patients who developed an NI had significantly higher percentages of CD71^+^CD235a^+^ cells (p = 0.021) and CD71^+^CD235a^+^CD45^+^ cells (p = 0.016), but not CD71^+^ cells (p = 0.113), than those who did not (**Figures 3D–F**). The percentage of CD71^+^CD235a^+^ cells was higher in non-survivors when compared with survivors [1.3(0.4,6.6) vs. 0.7(0.2, 1.3); p = 0.037] (**Figures 3G–I**).

### CD71^+^ Cells and CECs for Predicting NIs in Septic Patients

The Cox proportional hazard model was used to analyze the relationship between CECs and NIs (**Table 2**). The results show that the percentage of CD71^+^ cells (HR 1.06; 95% CI 1.02–1.11; p = 0.006), CD71^+^CD235a^+^ cells (HR 1.08; 95% CI 1.02–1.14; p = 0.006), and CD71^+^CD235a^+^CD45^+^ cells (HR 9.63; 95% CI 2.31–40.27; p = 0.002) were associated with increased incidence of NIs after adjusting for BMI, APACHE II score, pathogen isolates, IL-6, hemoglobin, serum creatinine, intubation, central venous catheterization, and durations of mechanical ventilation, central venous catheterization, and urinary tract catheterization. From the ROC curve analysis, 2.96 was the best cutoff value of CD71^+^ cells to predict NI development [area under the curve (AUC) 0.605, p = 0.067, sensitivity 66.7%, specificity 57.6%]. The best cutoff values of CD71^+^CD235a^+^ cells and CD71^+^CD235a^+^CD45^+^ cells to predict NI development were 2.08 (AUC 0.653, p = 0.021, sensitivity 40.7%, specificity 86.4%) and 0.25 (AUC 0.660, p = 0.016, sensitivity 37.0%, specificity 90.9%).

**Table 2:** Cox regression analysis and competing-risk regression analysis of CD71+ cells and CECs for predicting nosocomial infection and mortality in septic patients.

Variables	Unadjusted model			Adjusted model		
	HR	CI_95_	p value	HR	CI_95_	p value
^a^ **Nosocomial infection**						
** Cox regression analysis**						
Percentage of CD71^+^ cells	1.05	1.01~1.09	0.010	1.06	1.02~1.11	0.006
Percentage of CD71^+^CD235a^+^ cells	1.09	1.05~1.15	<0.001	1.08	1.02~1.14	0.006
Percentage of CD45^+^CD71^+^CD235a^+^ cells	6.34	2.15~18.69	0.002	9.63	2.31~40.27	0.002
**Competing-risk regression analysis**						
Percentage of CD71^+^ cells	1.05	1.01~1.09	0.011	1.06	1.02~1.09	<0.001
Percentage of CD71^+^CD235a^+^ cells	1.09	1.06~1.13	0.001	1.08	1.04~1.12	0.001
Percentage of CD45^+^CD71^+^CD235a^+^ cells	5.81	1.87~18.70	0.003	6.77	1.39~32.93	0.018
^b^30-day mortality						
**Cox regression analysis**						
Percentage of CD71^+^ cells	1.04	1.00~1.08	0.035	1.05	1.00~1.10	0.041
Percentage of CD71^+^CD235a^+^ cells	1.07	1.02~1.11	0.002	1.06	1.00~1.12	0.031
Percentage of CD45^+^CD71^+^CD235a^+^ cells	3.14	0.93~10.63	0.065	2.20	0.51~9.45	0.288
**Competing-risk regression analysis**						
Percentage of CD71^+^ cells	1.04	1.01~1.07	0.009	1.04	1.01~1.07	0.015
Percentage of CD71^+^CD235a^+^ cells	1.07	1.05~1.09	<0.001	1.06	1.01~1.10	0.011
Percentage of CD45^+^CD71^+^CD235a^+^ cells	2.91	1.10~7.74	0.032	2.06	0.90~4.75	0.089

CECs, CD71+ erythroid cells; HR, HR hazard ratio; CI, confidence interval.

a Admission variables included in this model were BMI, APACHE II score, SOFA score, pathogen isolates, IL-6, hemoglobin, serum creatinine, intubation, central venous catheterization, and durations of mechanical ventilation, central venous catheterization, and urinary tract catheterization.

b Admission variables included in this model were gender, chronic heart disease, APACHE II score, SOFA score, IL-6, hemoglobin, serum creatinine, intubation, central venous catheterization, and durations of mechanical ventilation.

**Figure 4:**
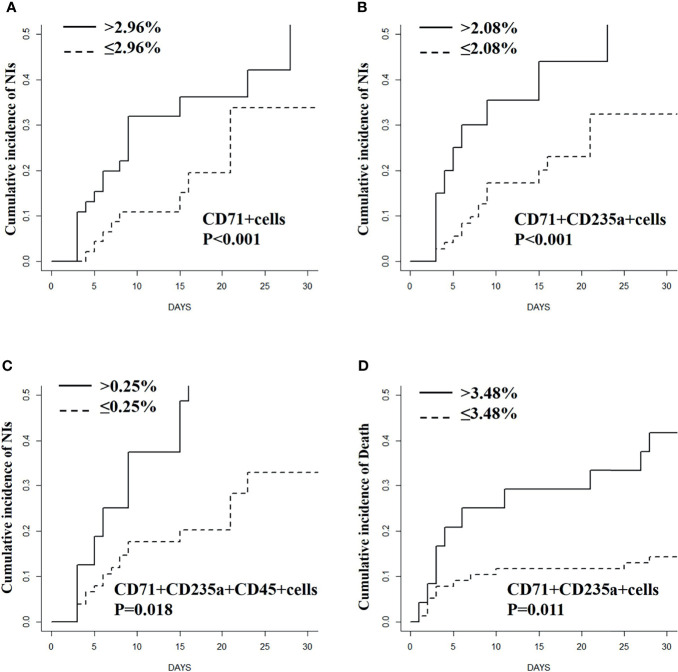
Cumulative incidence curves for nosocomial infections **(A–C)** and mortality **(D)** stratified based on the percentage of CD71^+^ cells and (or) CECs in septic patients.

In competing-risk regression analyses, the percentages of CD71^+^ cells (HR 1.06; 95% CI 1.02–1.09; p < 0.001), CD71^+^CD235a^+^ cells (HR 1.08; 95% CI 1.04–1.12; p = 0.001), and CD71^+^CD235a^+^CD45^+^ cells (HR 6.77; 95% CI 1.39–32.93; p = 0.018) were associated with NI development after adjusting for possible confounders (**Table 2**). According to cumulative incidence curves, for patients with high levels of CD71^+^ cells (>2.96%), CD71^+^CD235a^+^ cells (>2.08%), and CD71^+^CD235a^+^ CD45^+^ cells (>0.25%), the probabilities of developing an NI within the next 30 days were 52.8%, 66.6%, and 71.3%, whereas for patients with a low percentage (CD71^+^ cells ≤2.96%; CD71^+^CD235a^+^ cells ≤2.08%; CD71^+^CD235a^+^ CD45^+^ cells ≤0.25%), the probabilities were 33.8%, 32.4%, and 32.9%, respectively (all p < 0.05) (**Figures 4A–C**).

### CD71^+^ Cells and CECs for Predicting Mortality in Septic Patients

In Cox regression analysis, the percentages of CD71^+^CD235a^+^ CD45^+^ cells were not significantly associated with 30-day mortality after adjusting for confounders (HR 2.20; 95% CI 0.51–9.45; p = 0.288), while the percentage of CD71^+^ cells (HR 1.05; 95% CI 1.00–1.10; p = 0.041) and CD71^+^CD235a^+^ cells (HR 1.06; 95% CI 1.00–1.12; p = 0.031) could predict 30-day mortality outcome (**Table 2** and **Table S3**). From ROC curve analysis, 3.48% was the best cutoff value of CD71^+^CD235a^+^ cells to predict 30-day mortality (AUC 0.640, p = 0.046, sensitivity 42.9%, specificity 90.0%), while 6.05% was the best cutoff value of CD71^+^ cells (AUC 0.62, p = 0.068, sensitivity 47.6%, specificity 77.5%). In competing-risk regression analysis, the percentage of CD71^+^ cells (HR 1.04; 95% CI 1.01–1.07; p = 0.015) and CD71^+^CD235a^+^ cells (HR 1.06; 95% CI 1.01–1.10; p = 0.011) was associated with 30-day mortality after adjusting for possible confounding factors (**Table 2**). According to cumulative incidence curves, for patients with a percentage of CD71^+^CD235a^+^ cells more than 3.48%, the probability of mortality in 30 days was 14.3%, whereas for patients with a low percentage of the cells (≤3.48%), the probability was 52.9% (p < 0.001) (**Figure 4D**).

### Factors Associated With the Expansion of CD71^+^ Cells and CECs in Sepsis

Multiple linear regression was performed to detect independent factors associated with the expansion of CD71^+^ cells and CECs (**Table S4**). Potential variables included in the regression analysis were age, gender, comorbidities, the severity scores (SOFA and APACHE II), WBC, RBC, Hb, Scr, and cytokines (IL-2, IL-4, IL-10, TNF-α, and IFN-γ). The results showed that the levels of IL-6 and IFN-γ were positively associated with the percentage of CD71^+^ cells, CD71^+^CD235a^+^ cells, and CD45^+^ subset of CD71^+^CD235a^+^ cells in PBMCs, while IL-10 was negatively associated with them. Additionally, the levels of RBCs were negatively associated with the percentage of CD71^+^CD235a^+^ CD45^+^ cells (**Table S4**).

## Discussion

Immune paralysis is one of the main pathophysiological characteristics of sepsis. Due to the decreased ability to kill the invading harmful microorganisms, septic patients with immune paralysis not only find it difficult to recover from the primary infection but also have an increased susceptibility to nosocomial infections (2–4, 19, 20). In light of these findings, immunomodulation has been considered as a promising therapeutic strategy for sepsis (5, 21, 22). Consequently, biomarkers for selecting patients with immunosuppression and the potential targets of immunotherapy urgently remain to be explored.

CD71, CD235a, and CD45 are widely used markers for identifying various differentiation stages of erythroid lineage cells (8, 9). During the maturation of erythroid cells, the expression levels of CD71 first increased and then decreased. Mature erythrocytes have no nucleus and do not express CD71. However, the expression of CD235a continued to the mature erythrocyte stage. If the cells express both CD71 and CD235a, it indicates that they are in an immature state. The erythroid lineage cells were generally identified by the gradual loss of CD45, a pan leukocyte marker. Thus, CD45 was used as a marker of early-stage CD71^+^CD235a^+^ cells (CECs). Recently, studies found that both CECs and CD45^+^ CECs had unappreciated immunosuppressive functions (8, 9, 23–25). In animal and cell models, CECs and CD45^+^ CECs suppress T-cell activation and inhibit the production of pro-inflammatory cytokines which contribute to cancer development and human immuno-deficiency virus (HIV) infection (8, 9, 12). Additionally, the increased levels of CECs have been found in patients with cancer, anemia, and HIV (10, 12, 26). In patients with COVID-19, a robust expansion of CECs was also observed (27). Nevertheless, no study has been designed to investigate the frequency of CECs in adult septic patients, and the potential clinical significance of them.

The present study found that the frequency of CECs in septic patients was higher than that in ICU controls. Additionally, septic patients who developed an NI had higher frequency of CD71^+^ cells, CD71^+^CD235a^+^ cells, and CD71^+^CD235a^+^CD45^+^ cells when compared with those who did not develop an NI. The results of Cox proportional hazard regression and competing-risk regression analyses showed that all these cells were independent risk factors of NIs in sepsis. In the present cohort, CD71^+^CD235a^+^ cells can also be used to predict the 30-day mortality of sepsis. A recent study found that, in tumor-bearing mice, prevention of CEC accumulation decreased tumor growth (12). Studies also showed that CD71^+^ cell depletion decreased bacterial load in a mouse model of polymicrobial sepsis and mice with various bacterial infections (15, 26). Therefore, CECs may be a promising therapeutic target for sepsis.

Anemia is a common feature during sepsis (28). Our study found that the levels of RBC were negatively associated with the frequency of CD45^+^ CECs which indicates that anemia may contribute to the expansion of CECs. Erythropoietin (EPO) may be a mediator of CEC expansion caused by anemia in sepsis. Studies have illustrated that EPO can induce the expansion of highly proliferative early-stage CECs (CD45^+^ CECs), and neutralization of EPO prevents infection-related CEC accumulation (29, 30). In addition to anemia, many pro-inflammatory cytokines play crucial roles in the expansion of CECs (8, 10). IFN-γ stimulates CEC expansion by reducing RBC lifespan and increasing macrophage erythrophagocytosis (31). Other cytokines, including TNF-α, IL-1, and IL-6, lead to the expansion of CECs and enrichment of early-stage CECs by impairing erythropoiesis and aggravating anemia (8, 10). In the present study, IL-6 and IFN-γ were positively associated with the frequency of CECs. Interestingly, IL-10, an important anti-inflammatory cytokine in sepsis, was negatively associated with the frequency of CECs. Collectively, the present study suggested that the association existed in cytokine expression and CEC expansion among septic patients, but the interactions between them remains to be further investigated in the animal models.

Our study has some limitations. Firstly, the dynamic changes of the proportions of CECs in sepsis were not studied. Sustained high proportions of CECs may have a high predictive value for the adverse outcomes of sepsis. Secondly, there were 21 non-survivors in the present cohort and the 30-day mortality was 20.8%. The frequencies of CD71^+^ cells and CD71^+^CD235a^+^ CD45^+^ cells in non-survivors appeared to be higher than in survivors, although there was no statistical difference in some models. A relatively small number of non-survivors may lead to an inaccurately estimation of the prognostic value of these cells. Thirdly, although the Cox regression model and competing-risk regression model were used in the present study, the interaction or non-linear relationship between covariates and outcomes may not be fully considered (32). Finally, this study is a single-center study. The results should be validated prospectively in a multicenter trial.

## Conclusions

In conclusion, the present study found that CD71^+^ erythroid cells were expanded in adult septic patients and can serve as independent predictors of the development of NI and 30-day mortality. Low levels of RBCs and high levels of IL-6 and IFN-γ may contribute to the expansion of CD71^+^ erythroid cells in sepsis.

## Data Availability Statement

The raw data supporting the conclusions of this article will be made available by the authors, without undue reservation.

## Ethics Statement

The study was reviewed and approved by the Institutional Review Board of the First Affiliated Hospital of Wenzhou Medical University, Wenzhou, China (2019040), and was registered on the website of China Registered Clinical Trial Registration Center with No. ChiCTR1900024887. Informed consent was obtained from all participants prior to enrollment. The patients/participants provided their written informed consent to participate in this study.

## Author Contributions

GJZ designed the study; collected and analyzed the data; and contributed to writing this manuscript. DWJ and WCC collected and analyzed the data. XYC, WD, LWC, GLH, and BW helped with data analysis. YMY and ZQL designed and supervised the study and drafted the manuscript. All authors contributed to the article and approved the submitted version.

## Funding

This work was supported, in part, by grants from the National Natural Science Foundation of China (81871583, 81772112).

## Conflict of Interest

The authors declare that the research was conducted in the absence of any commercial or financial relationships that could be construed as a potential conflict of interest.

## Publisher’s Note

All claims expressed in this article are solely those of the authors and do not necessarily represent those of their affiliated organizations, or those of the publisher, the editors and the reviewers. Any product that may be evaluated in this article, or claim that may be made by its manufacturer, is not guaranteed or endorsed by the publisher.
