# Supplementary material for: CD71+ Erythroid Cell Expansion in Adult Sepsis: Potential Causes and Role in Prognosis and Nosocomial Infection Prediction
Source: Front Immunol. 2022 Feb 18;13:830025. doi: 10.3389/fimmu.2022.830025 (PMC8896534; doi:10.3389/fimmu.2022.830025)
Supplement: Supplementary file 1 [file DataSheet_1.docx]

**Supplementary Material**

**Figure S1. The proportion of CD71^+^CD235^+^ cells in peripheral blood mononuclear cells**

**Table S1. Baseline characteristics of critically ill patients with and without sepsis**

|  | Critically ill patients  with sepsis (n=101) | Critically ill patients  without sepsis (n=32) | P value |
| --- | --- | --- | --- |
| Age (year) | 65.0(56.0, 70.5) | 69.5(61.0, 76.5) | 0.188 |
| Gender (male) , n (%) | 57(56.4%) | 19(59.4%) | 0.467 |
| BMI, kg/m2 | 22.0(20.0, 25.0) | 22.0(18.5, 25.8) | 0.650 |
| Co-morbidities, n (%) |  |  |  |
| Diabetes | 22(21.8%) | 10(31.3%) | 0.195 |
| Chronic heart disease | 10(9.9%) | 3(9.4%) | 0.617 |
| Hypertension | 56(55.4%) | 21(65.6%) | 0.209 |
| Chronic liver disease | 9(8.9%) | 8(25.0%) | 0.077 |
| Chronic kidney disease | 13(12.9%) | 0(0%) | 0.090 |
| Cancer | 18(17.8%) | 2(3.1%) | 0.089 |
| APACHE II score，median (IQR) | 14.0(9.5, 18.0) | 13.5(10.3, 19.0) | 0.772 |
| Percentage of CD71^+^cells, median (IQR) | 2.9(1.4, 6.8) | 0.9(0.3, 2.1) | <0.001 |
| Percentage of CD71^+^CD235a^+^cells, median (IQR) | 0.9(0.3, 2.0) | 0.3(0.1, 0.7) | 0.001 |
| Percentage of CD45^+^CD71^+^CD235a^+^cells, median (IQR) | 0.10(0.05,0.21) | 0.04(0.01,0.08) | <0.001 |

Abbreviations: IQR: interquartile ranges; APACHE, Acute Physiology and Chronic Health Evaluation.

**Table S2. Pathogens identified from patients with nosocomial infection**

|  | PI (%) | UTI (%) | BSI (%) | CRI (%) |
| --- | --- | --- | --- | --- |
| Pathogen | n=20 | n=7 | n=3 | n=3 |
| 1. baumannii | 5 | 0 | 1 | 0 |
| S. maltophilia | 5 | 0 | 0 | 0 |
| P. aeruginosa | 4 | 0 | 0 | 0 |
| B. cepacia | 2 | 0 | 0 | 0 |
| Corynebacterium | 2 | 0 | 0 | 0 |
| E. coli | 1 | 0 | 0 | 0 |
| Serratia | 1 | 0 | 0 | 0 |
| Enterococcus | 0 | 3 | 0 | 1 |
| Staphylococcus | 1 | 0 | 2 | 1 |
| Candida | 1 | 7 | 0 | 3 |

PI: pulmonary infection; BSI: bloodstream infection; SSI/STI: surgical site and soft tissue infections; UTI: urinary tract infection

**Table S3. Baseline characteristics of survivors and non-survivors with sepsis**

|  | **Septic patients** | | |  |
| --- | --- | --- | --- | --- |
| **Variables** | **Total (n=101)** | **Survivors(n=80)** | **Non-survivors(n=21)** | **P value** |
| Age (year) | 64.5±1.2 | 64.1±1.4 | 66.3±2.6 | 0.301 |
| Gender (male) | 57(56.4%) | 40(51.3%) | 17(73.9%) | 0.013 |
| BMI, kg/m2 | 22(20.25) | 22(20,25) | 22(20, 25) | 0.520 |
| Percentage of CD71^+^cells | 2.9(1.4,6.8) | 2.7(1.0,5.8) | 4.0(1.5,9.0) | 0.130 |
| Percentage of CD71^+^CD235^+^cells | 0.9(0.3,2.0) | 0.7(0.2,1.3) | 1.3(0.4,6.6) | 0.037 |
| Percentage of CD45^+^CD71^+^CD235^+^cells | 0.1(0.0,0.2) | 0.1(0.0,0.2) | 0.1(0.1, 0.4) | 0.169 |
| Circulating cytokines (pg/ml) |  |  |  |  |
| IL-2 | 0.7(0.4,1.3) | 0.7(0.5, 1.3) | 0.6(0.4, 1.1) | 0.570 |
| IL-4 | 0.4(0.2,0.6) | 0.4(0.1, 0.5) | 0.5(0.2, 0.7) | 0.278 |
| IL-6 | 361.1(72.1,2971.3) | 249.9(64.5, 2971.3) | 2615.4(162.4, 17945.3) | 0.012 |
| IL-10 | 50.7(12.2,407.7) | 45.6(8.9, 407.7) | 76.7(17.3, 585.4) | 0.142 |
| TNF-α | 1.8(0.7,5.6) | 2.1(0.7, 5.6) | 1.2(0.5, 3.9) | 0.184 |
| IFN-γ | 1.6(0.5,3.1) | 2.0(0.6, 4.3) | 1.0(0.4, 3.0) | 0.211 |
| Co-morbidities, n(%) |  |  |  |  |
| Diabetes | 22(21.8%) | 19(24.4%) | 3(13.0%) | 0.248 |
| Chronic heart disease | 10(9.9%) | 4(5.1%) | 6(26.1%) | 0.003 |
| Hypertension | 56(55.4%) | 41(52.6%) | 15(65.2%) | 0.283 |
| Chronic liver disease | 9(8.9%) | 6(7.7%) | 3(13.0%) | 0.429 |
| Chronic kidney disease | 13(12.9%) | 8(10.3%) | 5(21.7%) | 0.148 |
| Cancer | 18(17.8%) | 13(16.7%) | 5(21.7%) | 0.576 |
| Infection sites, n(%) |  |  |  | 0.228 |
| Digestive tract | 32(31.7%) | 27(34.6%) | 5(21.7%) |  |
| Urinary tract | 27(26.7%) | 22(28.2%) | 5(21.7%) |  |
| Skin and soft tissue | 16(15.8%) | 9(11.5%) | 7(30.4%) |  |
| Respiratory tract | 7(6.9%) | 6(7.7%) | 1(4.3%) |  |
| Others | 19(18.8%) | 14(17.9%) | 5(21.7%) |  |
| Pathogen isolates, n(%) |  |  |  | 0.667 |
| Gram-negative bacteria | 23(22.8%) | 17(21.8%) | 6(26.1%) |  |
| Gram-positive bacteria | 9(8.9%) | 8(10.3%) | 1(4.3%) |  |
| Fungus | 2(2.0%) | 2(2.6%) | 0(0.0%) |  |
| Virus | 1(1.0%) | 1(1.3%) | 0(0.0%) |  |
| ^a^Other pathogens | 3(3.0%) | 3(3.8%) | 0(0.0%) |  |
| Mixed | 4(4.0%) | 2(2.6%) | 2(8.7%) |  |
| No | 59(58.4%) | 45(57.7%) | 14(60.9%) |  |
| ^b^SOFA score, median (IQR) | 7(5,9) | 6(4, 9) | 10(8, 12) | P<0.001 |
| ^b^APACHE II score |  |  |  |  |
| Overall, median (IQR) | 14(10,18) | 12(8,16) | 19(16, 24) | P<0.001 |
| <8 | 21(20.8%) | 21(26.9%) | 0(0.0%) |  |
| 9-13 | 26(25.7%) | 24(30.8%) | 2(8.7%) |  |
| 14-17 | 26(25.7%) | 21(30.8%) | 5(21.7%) |  |
| >17 | 28(27.7%) | 12(15.4%) | 16(69.6%) |  |
| Hemoglobin (g/L) | 103.0(86.5,116.0) | 105.5(89.8, 118.0) | 95.0(70.7, 108.0) | 0.015 |
| Red blood cells (×10^12/L) | 3.4(2.9,3.9) | 3.6(3.0, 3.9) | 3.2(2.7, 3.6) | 0.023 |
| White blood cells (×10^9/L) | 13.9(8.1,20.3) | 13.5(8.1, 18.5) | 16.1(8.1, 22.8) | 0.604 |
| Lymphocytes (×10^9/L) | 0.7(0.5,1.0) | 0.7(0.5, 1.0) | 0.7(0.5, 0.8) | 0.343 |
| Serum creatinine (μmol/L) | 155(87,276) | 116(75, 227) | 254(148, 324) | P<0.001 |
| Interventions, n(%) |  |  |  |  |
| Intubation | 27(26.7%) | 14(17.9%) | 13(56.5%) | P<0.001 |
| Duration of Mechanical ventilation | 0(0, 1) | 0(0, 0) | 1(0, 5) | 0.001 |
| Central venous catheterization | 73(72.3%) | 22(95.7%) | 51(65.4%) | 0.004 |
| Duration of Central venous catheterization | 4(0,7) | 4(0, 7) | 3(2, 12) | 0.144 |
| Urinary tract catheterization | 94(93.1%) | 73(93.6%) | 94(93.1%) | 0.705 |
| Duration of urinary tract catheterization | 6(3,11) | 6(4, 11) | 4(2, 10) | 0.273 |
| Nosocomial infection, n(%) | 27(26.7%) | 18(23.1%) | 9(39.1%) | 0.126 |

Abbreviations: IQR: interquartile ranges; APACHE, Acute Physiology and Chronic Health Evaluation; SOFA: Sequential Organ Failure Assessment.

^a^ Other pathogens are rickettsia, leptospira and plasmodium falciparum.

^b^ Scores were calculated within the first 24 h after the ICU admission using the value associated with the greatest severity of illness.

**Table S4. Results of multiple linear regression analysis of factors affecting the percentages of CECs in PMBCs**

| **Variables** | **Non-standardized coefficients** | | **Standardized coefficients** | **T** | **P value** |
| --- | --- | --- | --- | --- | --- |
|  | **B** | **Standard error** | **β** |  |  |
| **CD71^+^cells** |  |  |  |  |  |
| IL-6 | <0.001 | <0.001 | 0.375 | 2.446 | 0.016 |
| IL-10 | -0.002 | 0.001 | -0.335 | -2.359 | 0.021 |
| IFN-γ | 0.518 | 0.209 | 0.258 | 2.474 | 0.015 |
| **CD71^+^CD235a^+^cells** |  |  |  |  |  |
| IL-6 | <0.001 | 0.000 | 0.493 | 3.269 | 0.002 |
| IL-10 | -0.001 | 0.001 | -0.360 | -2.575 | 0.012 |
| IFN-γ | -0.009 | 0.045 | 0.260 | 2.531 | 0.013 |
| **CD71^+^CD235a^+^CD45^+^cells** |  |  |  |  |  |
| IL-6 | 1.272E-5 | 0.000 | 0.385 | 2.716 | 0.008 |
| IL-10 | -5.247E-5 | 0.000 | -0.302 | -2.302 | 0.024 |
| IFN-γ | 0.026 | 0.006 | 0.439 | 4.556 | <0.001 |
| RBC | -0.064 | 0.022 | -0.288 | 0.005 | 0.005 |

Abbreviations: CECs, CD71+ erythroid cells; IL, interleukin; IFN, interferon; RBC, red blood cells.
